# Spiral Annealing of Magnetic Microwires

**DOI:** 10.3390/s24196239

**Published:** 2024-09-26

**Authors:** Alexander Chizhik, Paula Corte-Leon, Valentina Zhukova, Juan Mari Blanco, Julian Gonzalez, Arcady Zhukov

**Affiliations:** 1 Department of Polymers and Advanced Materials, University of Basque Country, UPV/EHU, 20018 San Sebastian, Spain; paula.corte@ehu.eus (P.C.-L.); valentina.zhukova@ehu.eus (V.Z.); julianmaria.gonzalez@ehu.eus (J.G.); arkadi.joukov@ehu.eus (A.Z.); 2 Department of Applied Physics, EIG, University of Basque Country, UPV/EHU, 20018 San Sebastian, Spain; juanmaria.blanco@ehu.eus; 3 IKERBASQUE, Basque Foundation for Science, 48011 Bilbao, Spain

**Keywords:** soft magnetic materials, amorphous magnetic microwires, magnetic domains, magneto-optic Kerr effect, magnetic anisotropy

## Abstract

A preprocessing technique named “spiral annealing” was applied for the first time to magnetic microwires. In this process, the sample was arranged in a flat spiral shape during annealing, and subsequent measurements were conducted on the unbent sample with the induced stress distribution along and transverse to the sample. The research utilized both magnetic and magneto-optical methods. The anisotropy field magnitude in both the volume and surface of the microwire was measured, and for the first time, a direct correlation between the anisotropy field and the curvature of a spirally annealed microwire was established. Additionally, a connection between the type of surface domain structure and the degree of spiral curvature was identified. The preservation of the distribution of spiral annealing-induced magnetic properties both along and across the microwire is a key effect influencing the technological application of the microwire. The range of induced curvature within which a specific helical magnetic structure can exist was also determined. This insight links the conditions of spiral annealing to the selection of microwires as active elements in magnetic sensors.

## 1. Introduction

Amorphous soft magnetic materials, including ribbons and microwires, are used in a wide range of technological applications [1,2,3,4,5,6,7]. These materials are particularly promising as base components in magnetic sensors and actuators [8,9,10,11,12,13]. Their suitability for these purposes is due to their outstanding magnetic properties, reliable mechanical characteristics and the presence of a well-established and reliable manufacturing process.

On the other side, the magnetic microwires explored in this study are also of significant interest for fundamental research [14,15,16]. The wide variety of magnetic structures is particularly attractive, as it enables the study of both the rapid movement of isolated domain walls and the transformation of spiral magnetic structures. The cylindrical shape of magnetic wires allows the observation of specific magnetic properties, such as the giant magnetoimpedance (GMI) effect and magnetic bistability [1,17,18,19,20,21,22]. These effects are largely determined by the unique domain structure of the magnetic wires, which consists of an axially magnetized inner core and outer domain shell [17,23]. In particular, the significant GMI effect observed in magnetic microwires is directly linked to their high circumferential magnetic permeability [17,23,24,25,26,27]. At the same time, the magnetic bistability effect arises from the quick displacement of the solitary domain wall (DW) in the inner core [1,23].

The unique structure of a magnetic domain, which is an axially magnetized single domain surrounded by an outer magnetized shell, is formed under the action of stresses arising during the rapid solidification of the composite wire. This occurs because the microwire consists of a metal core encased in a glass coating. Thus, the primary source of internal stresses is the difference in thermal expansion coefficients between the metal core and the glass coating. These internal stresses are complex, with the axial component being the most significant [14,28]. In Co-rich microwires, the surface domain structure varies based on the chemical composition and preprocessing methods. The fundamental structure is helical, which can be further divided into elliptical and spiral structures [29]. Under specific conditions, circular or longitudinal magnetic structures can also be present.

There is a growing interest in the elastic properties of both magnetic and non-magnetic materials in contemporary research. Here is a short summary of topics that interest modern researchers: organic light-emitting diode displays on flexible substrates; flexible magnetic sensors based on spin-valve structures; bending strain-tailored properties of flexible spintronic devices; magnetic properties of magnetostrictive FeGa films; and Brillouin-based distributed fiber-sensing technology [30,31,32,33,34]. Despite the diversity of these research fields, they share a common theme—the correlation of flexible mechanical properties and various physical and chemical properties that have significant technological applications.

We focused on extended magnetic elements that can undergo various forms of mechanical manipulation, such as stretching, compression, torsion, and bending. This interest follows from our previous extensive research on this type of magnetic, long amorphous microwire.

We were particularly interested in both theoretical and experimental studies that examined magnetic domain structures and different types of domain boundaries in wires and tubes subjected to different forms of bending [35,36,37,38,39,40,41]. The new concept of “Curvilinear Magnetism” signifies that this field now integrates theoretical advances with novel and unexpected technical solutions. The idea of curvilinear geometry-induced effective anisotropy was especially important for us to note. In microwires, the issue of induced anisotropy has long been a research focus [33,42,43,44,45,46,47], and this new approach to controlling it caught our attention.

We also focused on extended flat objects where the relationship between tension and compression during bending was thoroughly studied [34,48].

Extensive research on magnetic wires under axial tension, both during annealing preprocessing and in practical studies, indicated that the incorporating of amorphous microwires into the broader concept of “Curvilinear Magnetism” could be fruitful. Previously, the effect of bending stresses on the magnetic properties of microwires had been studied only sporadically, and we aimed to address this gap. Our two pioneering studies on this topic [49,50] produced promising results. This led us to the concept of applying mechanical stress in the form of a flat spiral.

In this study, we focused on a new preprocessing technique that we named spiral annealing. A spiral, in essence, is a bend with a continuously changing geometrical curvature. This configuration interested us because it offers the potential to create extended samples with distributed magnetic properties. Our extensive experience in studying flexible glass-coated microwires led us to believe that these materials are particularly well suited for investigating the effects of mechanical bending on magnetic properties. An innovative idea we propose in our recent studies [49,50] is that after annealing in a bended configuration, the stress distribution induced along the sample’s length remains intact even after the sample is unbent.

Traditional microwire studies focus on creating a series of specific samples annealed under constant stress. This means that the entire spectrum of possible induced magnetic structures is not created in preparation for the experiments. In our case, the continuous and gradual change in properties in a spirally annealed sample provides a unique opportunity not to miss any essential details that could be decisive when selecting a specific microwire as the active element in a sensor.

Further motivation came from recent studies on the Matteucci effect and GMI effect in bent microwires [51,52]. Within the broader scope of this research, mechanically bending magnetic wires, followed by annealing to lock off the induced changes, introduces new and innovative possibilities for their technical applications.

To conduct the studies, we employed what we considered the most suitable combination of research methods, selecting those most appropriate to obtain the desired results. These included the fluxmetry method [53], the magneto-optical Kerr effect method [54] and the Sixtus–Tonks method [55].

## 2. Experimental Details

We investigated a glass-coated microwire with a chemical composition of Co_64.04_Fe_5.71_B_15.88_Si_10.94_Cr_3.4_Ni_0.03_, featuring a metallic core diameter of 94.6 μm and a total diameter of 126 μm, including the glass coating. The wire was prepared using the Taylor–Ulitovsky technique and had a length of 18 cm. Originally, the sample displayed a uniform distribution of magnetic properties along its length. The sample was annealed at a temperature of 300 °C for 45 min.

**Figure 1:**
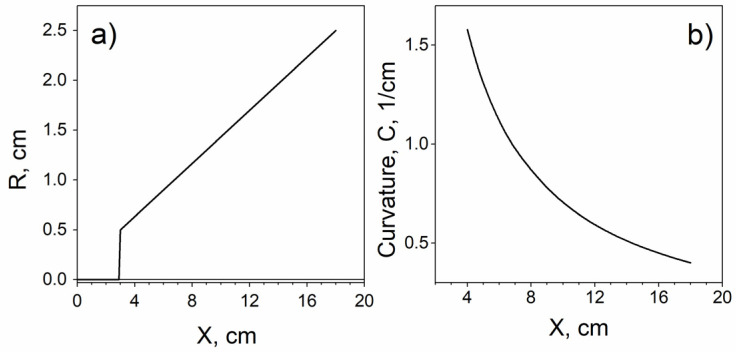
Dependence of radius (**a**) and curvature (**b**) of the annealed sample on the distance X from the sample edge.

We selected the flat spiral shape as the optimal configuration for achieving distributed magnetic properties both along and across the sample. In this study, we used the simplest version of the spiral, where the radius was directly proportional to the length (Figure 1). The bending of the spiral started away from the edge of the sample, allowing us to thoroughly examine the transition from the unbent section to the bent part. Specifically, the bending began 3 cm from the sample’s edge. Between 3 cm and 4 cm, there was a transition region where the spiral’s curvature became constant. Beyond 4 cm, the curvature was assumed to remain consistently inversely proportional to the radius. As will be shown later, the induced changes in magnetic properties still occur even at the end of the spiral, where the geometric bending tends to zero.

**Figure 2:**
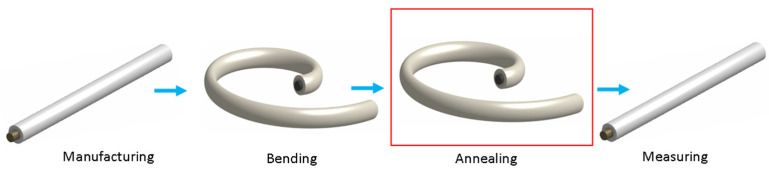
The schematic picture of sample preprocessing.

After the spiral annealing (the furnace in which the annealing took place is indicated by a red box in the Figure 2), the sample was straightened (as shown in Figure 2) and analyzed using the fluxmetric, magneto-optical, and Sixtus–Tonks methods.

The hysteresis loops were obtained using the fluxmetric technique, previously utilized for characterizing magnetically soft microwires and described in detail in [53]. This method involves a thin solenoid that produces a rather uniform axial magnetic field. The fluxmetric technique allows for the measurement of hysteresis loops up to H = 3 kA/m. To characterize the spiral-annealed microwires, which may display hysteresis loops of different shapes in various regions, a short pick-up coil (2 mm in length) was used. This setup allowed us to measure hysteresis loops across different longitudinal sections of the microwire by moving the short pick-up coil along its length. The magnetization reversal process in the surface region of the microwire was investigated using a MOKE (magneto-optical Kerr effect) loop tracer. Polarized light emitted from a laser was reflected from various locations on the microwire’s surface and then directed to a detector. The size of the MOKE spot was about 1 mm. The angle of the rotation of the polarization plane was proportional to the magnetization in the microwire’s surface layer.

The MOKE technique was also used to capture contrast images of surface magnetic domain structures. Kerr microscopy utilizes the magneto-optical Kerr effect to visualize magnetic domain structures locally in opaque magnetic materials. This optical technique enables the real-time, non-destructive analysis of the sample. We used a modern wide-field Kerr microscopy setup capable of achieving longitudinal Kerr geometries by employing obliquely incident light. This setup allows for the observation of the in-plane component of the magnetization. Although the surface of the microwire is not flat, we selected a surface area with a minimal curvature variation for the experiment, allowing us to disregard curvature effects as a first approximation. For our measurements, we adapted a wide-field MOKE microscope using a commercially available optical microscope (Carl Zeiss, Oberkochen, Germany), which we utilized for non-planar magnetic objects [54].

To analyze the domain wall (DW) structure at different locations in the studied microwire, we employed the Sixtus–Tonks method [55]. Rectangular magnetic field pulses, generated by a long solenoid, were used to displace the domain wall. The movement of the DW created peaks of electromotive force (EMF) in the secondary coil. By examining the shape of such types of EMF peaks, we inferred the structure of the DW.

## 3. Results and Discussion

**Figure 3:**
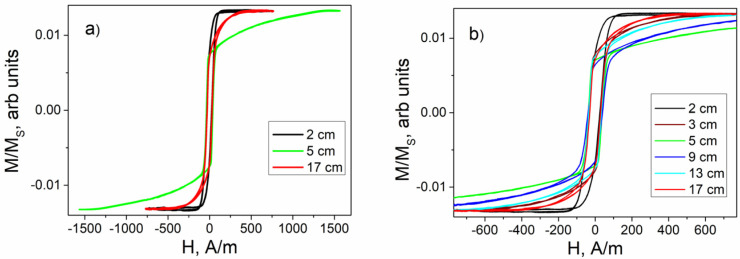
Fluxmetry hysteresis loops obtained at different points X along the length of the sample in long (**a**) and shorter (**b**) fields. Around a location of 5 cm (green line), saturation occurs at the maximum field value of about 1500 A/m (**a**). In this region of the spiral, the curvature is maximal. In short fields (**b**), one can see how the hysteresis loop becomes flatter in the middle part of the spiral (locations 9 cm, 13 cm).

**Figure 4:**
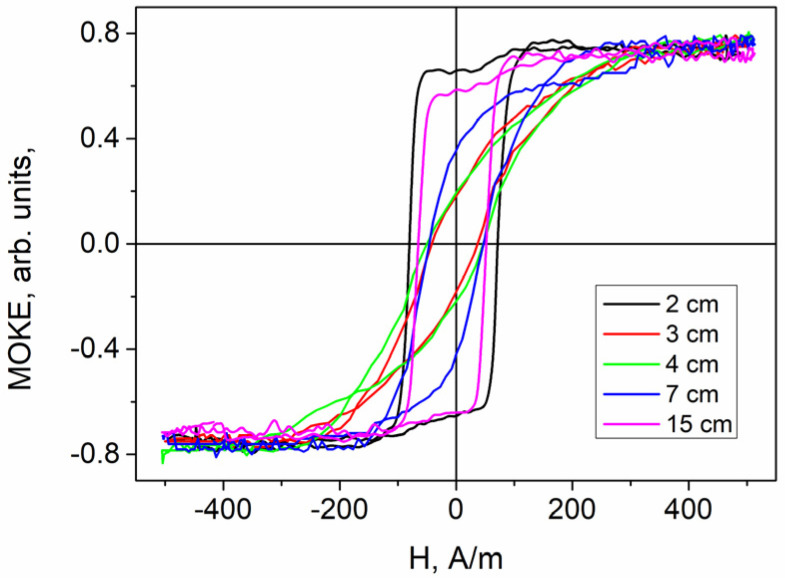
MOKE hysteresis loops obtained in different locations X along the sample length. The hysteresis loops obtained at locations of 2 cm and 15 cm have a rectangular shape, which indicates a surface Barkhausen jump. The loops obtained at locations of 3 cm, 4 cm and 7 cm have a smoother shape, which indicates the presence of a surface helical structure.

As an initial study, we obtained magnetic and magneto-optical hysteresis loops. Figure 3 and Figure 4 display the magnetic and MOKE hysteresis loops, respectively. The experimental setup involved moving a secondary coil (using the fluxmetry method) or a laser spot (using the MOKE method) along the length of the extended samples and fixing the secondary coil or laser spot at a specific location X. This approach resulted in magnetic and MOKE hysteresis loops of varying shapes, depending on the distance X from the sample’s edge. During the spiral annealing process, the curvature at each point of the sample changed smoothly along the sample length (Figure 1).

Figure 3a displays the magnetic hysteresis for the three most significant locations, recorded in a sufficiently large magnetic field, when the magnetization reached saturation. Figure 3b details the transformation of magnetic loops observed as the secondary coil was moved along the sample from one edge to the other.

**Figure 5:**
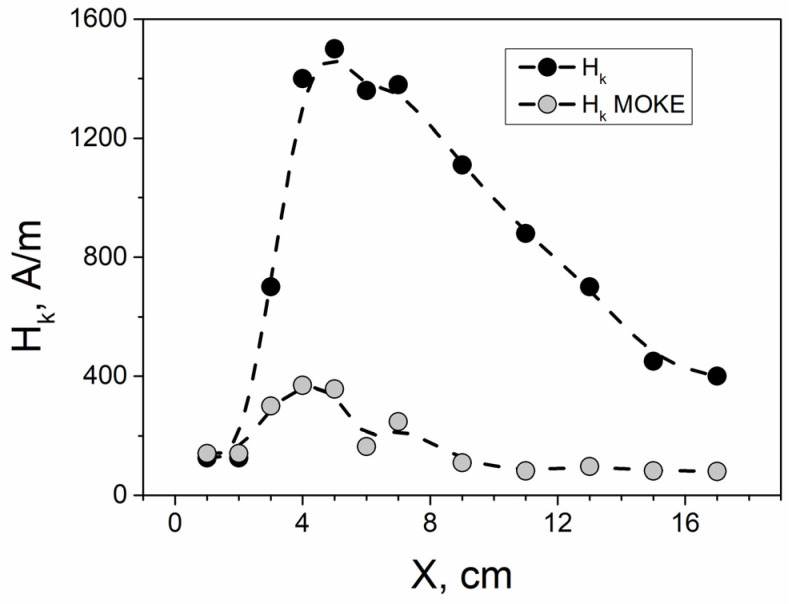
Dependence of the anisotropy field values H_k_ on X location. The black dots were extracted from magnetic measurements, while the gray dots were extracted from the MOKE experiments. During annealing the spiral formally started at point X = 3 cm and was reliably determined at point X = 4 cm. The peak of the anisotropy field value was observed at point X = 5 cm for volume and at point X = 4 cm for surface measurements.

When analyzing the results, we focused on an important parameter, the anisotropy field (H_k_). The anisotropy field value was determined from the shape of the hysteresis curves, using the method described in article [56]. The results of the analysis are shown in Figure 5.

The transformation of the MOKE loop (Figure 4) was also observed when the laser reflection point was moved along the sample length. This transformation was also analyzed based on the observed variation in the anisotropy field magnitude. Figure 5 illustrates the dependence of the anisotropy field magnitude on the X value, indicating the laser beam reflection point’s location.

The first essential detail is the significant difference in the absolute magnitudes of the anisotropy field derived from the magnetic (Figure 3) and MOKE (Figure 4) hysteresis loops, although the dependences on H_k_ themselves are quite similar. This effect of the difference between the bulk and surface anisotropy fields is observed for the first time. In the first approximation, it can be explained within the framework of the model proposed in the article [57]. This study proposes and substantiates a radial distribution of the anisotropy field, which decreases from the axis of the microwire towards its surface.

The obtained dependence of the anisotropy field on distance X can be divided into three basic parts. The first part is the initial part of the sample, before the beginning of the spiral. It starts from the edge of the sample and ends at a distance of 3 cm from the edge of the sample. This region is characterized by a hysteresis loop shape close enough to a rectangular one and a small value of the anisotropy field. We observed this type of magnetic behavior in some Co-rich microwires, preliminarily annealed without stress [58].

The second short section could be characterized as transitional. It spans from the point where the spiral formally begins (X = 3 cm) to the point where the curvature of the spiral can be reliably determined (X = 4) cm.

The final third section, starting at X = 4 cm and extending to the end of the sample (X = 18 cm), was of particular interest to us. In this region, we observed a smooth variation in the anisotropy field, corresponding to a gradual, continuous change in the sample’s curvature during the spiral annealing.

**Figure 6:**
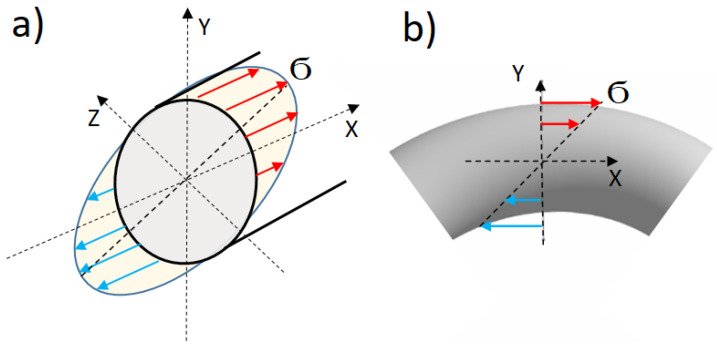
Distribution of bending stress in volume of sample (**a**) and in cross-section (**b**). Red and blue arrows show the direction of tensile and compressive stress, respectively. The stress value changes both in the sample volume and on the sample surface. In the case of spiral annealing, the absolute value of the induced stress decreases with increasing spiral radius (decreasing curvature).

Our analysis of the results is based on three fundamental ideas. The first idea addresses the relationship between the internal induced stress and the curvature of a long, stressed sample. The second idea explores the correlation between the induced anisotropy and the induced stress. The third idea emphasizes that in a sample subjected to bending, there is an internal, cross-sectional, spatial distribution of mechanical stress (Figure 6).

The fourth idea involves our concept of the continuous change in magnetic properties within an extended sample, which we are developing in our latest works [49,50]. This new conceptual approach allows, under certain conditions, a transition from studying a wide array of samples with varying properties to focusing on one or two long samples that exhibit a continuous variation in several interrelated properties. This is demonstrated in our current experiments, where the geometric curvature of the sample changes simultaneously with the anisotropic properties.

Regarding the mechanical stress, the bending stress consists of tensile stress along the outer region of the microwire and compressive stress along the inner region of the microwire, as illustrated in Figure 6. No stress is present along the neutral axis. According to Stoney’s fundamental formula [59,60], which first demonstrated the relationship between the mechanical stress and curvature of a bending object, the amplitude of the stress Ϭ applied to the wire cross-section can be determined using the following formula:

Ϭ = −E∙y/r = −E∙y∙C (1)

where E is the Young’s modulus of the wire, y is the distance to the neutral axis, r is the radius of curvature and C is the curvature.

According to this formula, Ϭ is positive when the distance y is negative (i.e., the outer region of the microwire), Ϭ is negative when y is positive (i.e., the inner region of the wire). Thus, the overall deformation in the wire’s volume is balanced, as the sample experiences equal amounts of tensile and compressive stress. However, detailed calculations [61] and experiments with magnetic impedance [52] have shown that the effects of tensile stress and compressive stress are not perfectly opposite. Consequently, the tensile stress tends to dominate in the curved sample.

From this reasoning, the key point for us is that in a curved element, stress is inversely proportional to the radius of the bending and directly proportional to the curvature. In our case of spiral annealing, this implies that a gradual decrease in the sample’s curvature along its length results in a corresponding gradual decrease in the internal stress.

The stress-induced anisotropy field H_k_ can be expressed using the following well-known formula [62]:

H_k_ = 3λϬ/M_S_ (2)

where Ϭ represents the stress, λ is the saturation magnetostrictive constant and M_S_ is the saturation magnetization.

**Figure 7:**
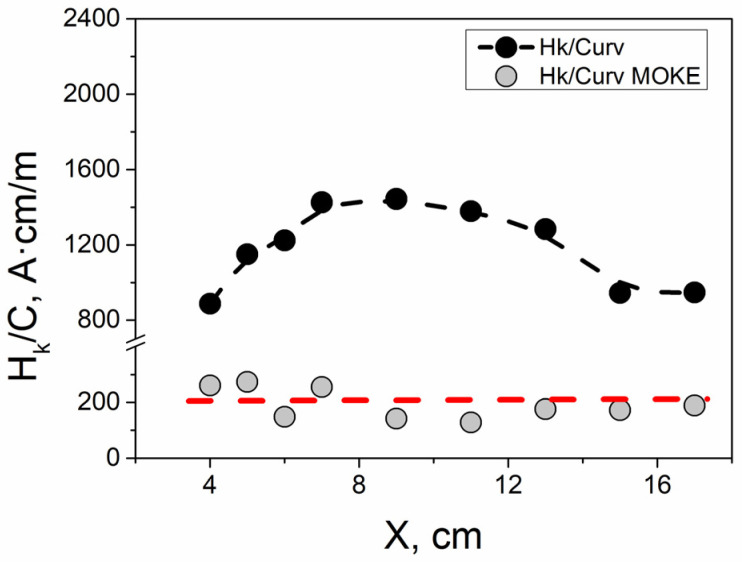
Dependence of the anisotropy field normalized to the curvature values H_k_/C on X location. The black dots were extracted from magnetic measurements, while the gray dots were extracted from the MOKE experiments. The red dashed line demonstrates that the magnitude of the surface anisotropy field is directly proportional to the geometric curvature of the sample.

Given the clear relationship between the radius and the curvature of the bended sample, we can combine Equations (1) and (2) to establish a direct dependence of the anisotropy field on the curvature of the sample that has undergone spiral annealing. There should be a linear relationship between H_k_ and C, assuming all other parameters in the equations remained constant during the experiment. To verify this, we constructed the H_k_/C(X) dependence for the cases of the fluxmetric and MOKE experiments (Figure 7).

For the MOKE measurements, the resulting dependence is nearly constant. In the case of fluxmetric measurements, although the points do not align precisely with the constant line, they fall within a relatively narrow range of values. This suggests a real relationship between the anisotropy field and the curvature.

The difference between the dependencies observed in the fluxmetric and MOKE experiments is due to the presence of a radial stress distribution resulting from spiral annealing. In volumetric hysteresis loops, this stress distribution is averaged over the entire cross-section. In MOKE experiments, however, we examine a narrow surface layer of the sample, where the stress is relatively uniform in magnitude.

The measurement error of magnetic and MOKE measurements was determined by the digitalization of the hysteresis loops, specifically by the number of points per hysteresis loop. In magnetic measurements, this number was 250 points per loop, and in MOKE it was 180 points per loop. As a result, keeping in mind also the averaging process applied, the measurement error of magnetic field measurement in the two experimental methods was about 15 A/m. It should be mentioned that only a single sample was investigated in this study.

**Figure 8:**
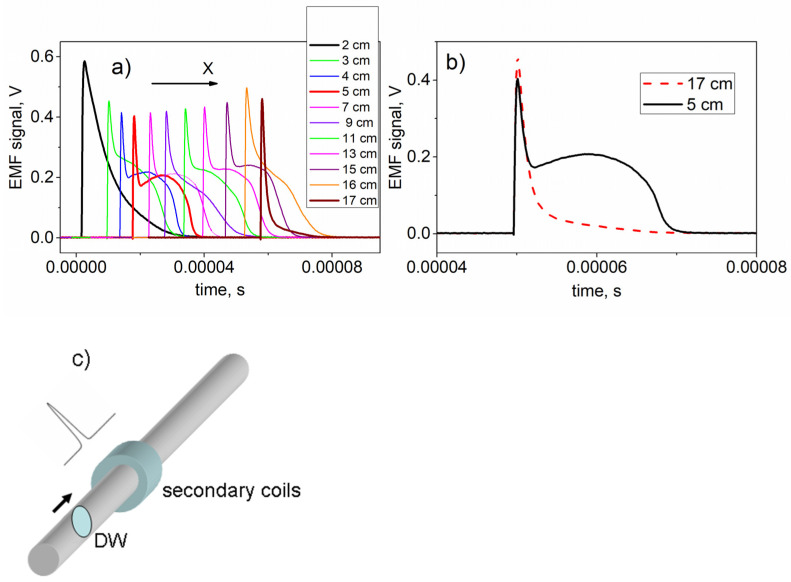
Experimental results obtained using the Sixtus–Tonks technique. (**a**) EMF peaks corresponding to different location of measurement X. The transformation of the peak shape is observed depending on location X. (**b**) Comparison of peaks obtained in locations X = 5 cm and X = 17 cm. (**c**) Schematic picture of the experiment. The black arrow shows the direction of DW motion. At locations of 2 cm and 17 cm (**a**), single sharp peaks are observed, corresponding to the movement of single compact domain boundaries. The width of the peaks depends on the degree of inclination of the isolated domain wall. An additional wide peak corresponds to the running of the helical wall shown in Figure 9b.

To gain a deeper understanding of how induced stress distribution affects the magnetic properties of the microwire, we conducted experiments using the Sixtus–Tonks technique. Specifically, we analyzed the shape of the EMF signal as the domain wall traversed a narrow secondary coil. As previously demonstrated [16], the shape of this signal reflects the structural characteristics of the domain wall. During the experiment, we were able to obtain the EMF signals from various locations within the sample. Figure 8a shows the successive transformation of the EMF peaks as the distance X measured from the edge of the sample was changed.

Let us focus on the key aspects of the series of EMF peaks obtained. It is known that a simple single EMF peak corresponds to the motion of a single domain wall through a secondary coil. The width of the EMF peak, among other factors, depends on the width of the domain wall. In Figure 8a, a single EMF peak is observed in two locations: in the initial region of the sample, before the beginning of the spiral (X = 2 cm), and in the final region, where the bend is small (X = 17 cm). The width of these EMF peaks differs, which we logically attribute to the variation in the width of the domain wall. In our case, this difference is due to the varying inclination of the domain wall, as will be shown below.

Between these two extreme locations (X = 2 cm and X = 17 cm), the peak shape becomes more complex. One part of the EMF peak retains the same sharp feature as before (single peak), while the wider part additionally arises. As the measurement location moves along the length of the sample, the proportion of these two components of the overall peak changes.

It is important to note that this type of double-peak signal has not previously been observed in the studies of magnetic microwires. To illustrate that a sharp single peak is part of a double peak, we overlaid two peaks measured at the positions of X = 5 cm and X = 17 cm (Figure 8b). As shown, the sharp single peak obtained at X = 17 cm perfectly aligns with the first peak recorded at X = 5 cm. Therefore, we conclude that the initial part of the complex magnetic structure, responsible for the double peak at X = 5 cm, is indeed a single domain wall with a slight inclination from the transverse direction.

**Figure 9:**
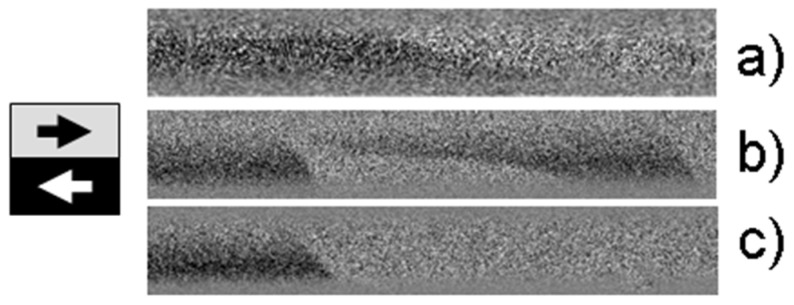
Surface domain structures observed in studied sample. (**a**) X = 2 cm, (**b**) X = 5 cm, (**c**) X = 17 cm. Black–white arrows show the direction of the magnetization in the domains. (**a**,**c**) show images of single surface domain walls with different inclinations from the transverse direction. (**b**) shows a combination of a single elliptical domain wall and a helical domain wall.

To further clarify the structure responsible for the domain formation that produces this type of EMF peak, we present MOKE microscopic images of the surface domain structure observed at the corresponding locations X = 2 cm, 5 cm and 17 cm (Figure 9). As anticipated, the locations X = 2 cm (Figure 9a) and X = 17 cm (Figure 9c) correspond to the isolated domain walls with varying deviation angles. A greater deviation from the transverse axis (X = 2 cm) is found at the location that was not subjected to bending during annealing. The smaller deviation occurs at the opposite edge of the sample (X = 17 cm), which experienced only slight bending during annealing. Finally, the region where the double peak was observed corresponds to a domain structure comprising a single domain wall and a short helical structure. The motion of this helical structure results in the second broad peak in the EMF signal.

Another important detail related to spiral annealing needs to be highlighted. This detail is evident in the experiments shown in Figure 8 and Figure 9 and is related to the transversal stress gradient across the sample, transitioning from tensile to compressive stress. This gradient, or radial stress distribution, explain the asymmetry in the domain structure within the region of the sample subjected to spiral annealing. Notably, the helical structure depicted in Figure 9b forms in the area where the tensile stress was at its peak during annealing (indicated by red arrows in the Figure 6). This suggests that tensile stress facilitates the formation of the domain structure, while compressive stress suppresses it. Thus, this interplay between tension and compression is responsible for the observed helical domain structure. When the bending during annealing is absent or minimal, both sides of the sample are equivalent in the matter of domain formation, as seen in Figure 9a,c.

The various forms of volume and surface hysteresis loops provide valuable information that helps draw conclusions about the potential applications of the microwires studied. A comparative analysis of hysteresis loops, EMF peaks and surface domain structure images appears to be the most effective approach.

As is well known, there are two main types of sensors that utilize microwires as active elements. The first type is based on the rapid movement of a domain wall (the Barkhausen jump), while the second type is based on the magneto-impedance dependence on the external magnetic field (the GMI effect).

A combined analysis of the magnetic and MOKE hysteresis, along with the EMF peak recorded at the 2 cm mark (black line in Figure 3, Figure 4 and Figure 8) and the image in Figure 9a, revealed the following: both on the sample’s surface and likely within the sample’s volume, a single domain wall moves rapidly. It is important to note that this domain wall significantly inclines from the transverse direction, characterizing it as elliptical. The 17 cm location also appears suitable for such sensors. Although the volume hysteresis curve is not perfectly rectangular (Figure 3, red lines), the shape of the EMF peak (Figure 8a, brown line) and the domain structure image confirm the presence of a rapidly moving single compact domain wall. Additionally, the wall’s minimal deviation from the transverse direction (Figure 9c) and the narrower EMF peak suggest this section of the sample is more favorable for use in Barkhausen-based sensors than the region of the sample close to the 2 cm location.

Regarding GMI-based sensors, the following can be noted: the magnitude of the GMI effect is directly linked to the transverse magnetic susceptibility, which is influenced by the type of surface magnetic structure. It is well known that the helical magnetic structure is well suited to provide a high level of transverse susceptibility. Specifically, there is an angular sector of the helical structure, around 60 degrees from the axial direction, which results in the maximum susceptibility. In this context, the segment of the sample between 4 cm and 15 cm is suitable for use in GMI sensors. A closer examination of Figure 8a allows for the identification of more distinct areas. The presence of a compact helical structure is essential, and the locations at X = 5 cm and X = 15 cm correspond to a more pronounced, isolated second wide EMF peak associated with the helical structure. This suggests that these locations (X = 5 cm and X = 15 cm) in the spiral-annealed microwire may be the most optimal for GMI-based sensors.

It is also known that the value of the magnetic field at the peak of the GMI effect directly correlates with the value of the surface anisotropy field of the microwire. As demonstrated in this paper, there is a direct relationship between the magnitude of the surface anisotropy field and the geometric curvature. GMI sensors should meet specific requirements regarding the peak field value and the range of operating fields. Therefore, by analyzing the hysteresis loops obtained from the spirally annealed sample, we can identify spatial locations with predicted magnetic field values corresponding to the maximum of the GMI effect.

The helical domain structure depicted in Figure 9b was observed between the 4 cm and 17 cm locations. Its appearance exhibited a clear threshold behavior. It is important to note that the domain structure was analyzed in a straightened sample where mechanical stress, induced during the spiral annealing process, was distributed. Under these conditions, the sharp increase in the internal stresses around the X = 3 cm point, where the spiral begins, causes the helical structure to become energetically comparable to the elliptical structure. At the point of X = 17 cm, the helical structure loses its stability in a similarly threshold-like manner.

The moment of the helical structure’s formation varied as the observation point shifted along the sample. This variation explains the changes in the peak shapes shown in Figure 8. The second, broader peak became more prominent when the helical structure emerged, with a slight delay relative to the appearance of the elliptical structure (for example, for the point of X = 5 cm).

Regarding the optimization of sensors using curved microwires based on the Matteucci and GMI effects, several key points can be highlighted. In the present study, we performed a preliminary identification of the domain structure in curved microwires, determining the range of induced curvatures where various types of spiral structures can form. This zone shows a high probability of achieving a strong Matteucci effect [51]. Additionally, we established a relation between the anisotropy field and the curvature induced by the bending stress. As a result, our work addresses a missing link in the understanding of how the GMI effect operates under bending stress [52].

This study on the effects of spiral annealing marks one of the initial steps toward utilizing microwires with properties distributed along their length. In the future, we plan to expand this research. First, we will apply this method to Fe-rich microwires, which also could be used as active elements in magnetic sensors. Additionally, we will explore flat and volumetric spirals with more complex curvature–radius relationships. These new geometric configurations are expected to produce novel and promising magnetic domain structures. Special attention will be given to the rate of change in the induced stress gradient along the length of the microwire, as a steep spatial gradient could result in more abrupt changes in surface and volume magnetic structures. This will lay the base for a deeper understanding of magnetic sensor operation.

It should be noted that the limitation of the spiral annealing method is the downside of its advantage. The same could be said about the challenges that arose during this study. The result of using the spiral annealing method is the creation of a long sample with distributed properties. On the other hand, to perform comparative GMI studies, it is necessary to cut a long sample into a series of small pieces. This irreversible cutting process does not allow us to return to the subsequent clarifying magnetic and MOKE studies. In this situation, we need to return to the beginning of the spiral annealing cycle.

## 4. Conclusions

In the context of developing samples with magnetic properties that vary continuously along their length, we introduced a new method of preprocessing called spiral annealing. Before annealing, the sample was arranged in a flat spiral shape with a predetermined curvature profile along its length. After annealing, the sample, which retained the induced mechanical stress, was straightened and analyzed. A gradient of internal stresses, ranging from tension to compression, was present across the sample’s cross section, with the stress gradient smoothly varying along its length. Magnetic and magneto-optical studies revealed a transformation in both the magnetization reversal process and the domain structure as the measurement location shifted along the sample. Notably, a gradual change in the anisotropy field was observed both within the microwire and on its surface. Analysis of the results indicated a linear relationship between the local anisotropy field and the local geometric curvature of the sample. The changes in the domain structure were found to correlate with the stress distribution induced in the sample. In certain regions of the microwire, a complex domain structure with a helical inclusion emerged due to the interplay between tension and compression.

## Data Availability

Data is contained within the article.
